# An RTM-GWAS procedure reveals the QTL alleles and candidate genes for three yield-related traits in upland cotton

**DOI:** 10.1186/s12870-020-02613-y

**Published:** 2020-09-07

**Authors:** Junji Su, Caixiang Wang, Qi Ma, Ai Zhang, Chunhui Shi, Juanjuan Liu, Xianliang Zhang, Delong Yang, Xiongfeng Ma

**Affiliations:** 1 Gansu Provincial Key Laboratory of Aridland Crop Science, College of Life Science and Technology, Gansu Agricultural University, Lanzhou, 730070 China; 2 Cotton Research Institute, Xinjiang Academy of Agricultural and Reclamation Science, Shihezi, 832000 China; 3 State Key Laboratory of Cotton Biology, Institute of Cotton Research of Chinese Academy of Agricultural Sciences, Anyang, 455000 China; 4 School of Agricultural Sciences, Zhengzhou University, Zhengzhou, 450001 China

**Keywords:** Upland cotton, Yield trait, RTM-GWAS, QTL alleles, Candidate genes

## Abstract

**Background:**

Cotton (*Gossypium* spp.) fiber yield is one of the key target traits, and improved fiber yield has always been thought of as an important objective in the breeding programs and production. Although some studies had been reported for the understanding of genetic bases for cotton yield-related traits, the detected quantitative trait loci (QTL) for the traits is still very limited. To uncover the whole-genome QTL controlling three yield-related traits in upland cotton (*Gossypium hirsutum* L.), phenotypic traits were investigated under four planting environments and 9244 single-nucleotide polymorphism linkage disequilibrium block (SNPLDB) markers were developed in an association panel consisting of 315 accessions.

**Results:**

A total of 53, 70 and 68 significant SNPLDB loci associated with boll number (BN), boll weight (BW) and lint percentage (LP), were respectively detected through a restricted two-stage multi-locus multi-allele genome-wide association study (RTM-GWAS) procedure in multiple environments. The haplotype/allele effects of the significant SNPLDB loci were estimated and the QTL-allele matrices were organized for offering the abbreviated genetic composition of the population. Among the significant SNPLDB loci, six of them were simultaneously identified in two or more single planting environments and were thought of as the stable SNPLDB loci. Additionally, a total of 115 genes were annotated in the nearby regions of the six stable SNPLDB loci, and 16 common potential candidate genes controlling target traits of them were predicted by two RNA-seq data. One of 16 genes (*GH_D06G2161*) was mainly expressed in the early ovule-development stages, and the stable SNPLDB locus (LDB_19_62926589) was mapped in its promoter region.

**Conclusion:**

This study identified the QTL alleles and candidate genes that could provide important insights into the genetic basis of yield-related traits in upland cotton and might facilitate breeding cotton varieties with high yield.

## Background

As a main industrial raw material, cotton (*Gossypium* spp.) fiber plays an important role in daily life and the world’s textile industry [1, 2]. Among the planting cotton, upland cotton (*Gossypium hirsutum* L.) is the largest cultivated species and accounts for more than 90% of cotton yield in the world [3]. Cotton fiber yield is one of the key target traits, and improved fiber yield has long been thought of as an important objective in the breeding procedures and production [4]. For fiber yield of a plant, its component factors contain boll number (BN), boll weight (BW) and lint percentage (LP), and the three traits are controlled by a sequence of quantitative trait loci (QTL). In the last decades, abundant QTL for cotton yield-related traits had been detected via linkage mapping method, and the QTL mapping results were summarized in cotton [5]. Over the past 5 years, a lot of significant single-nucleotide polymorphisms (SNPs) associated with fiber yield component traits have been identified by using genome-wide association studies (GWAS) methods in upland cotton [1, 4, 6–9]. These GWAS findings laid a good foundation for deciphering the genetic basis underlying cotton yield-related traits. However, the detected QTL for the target traits still remained limited, because only a handful of major QTL were identified through the conventional GWAS procedures.

The inchoate GWAS methods had a lot of trouble lowering the false-positive rate, which affected the identification accuracy of the associated loci [10, 11]. To enhance the detection efficiency of the authentic QTL, three GWAS procedures, including the structured association analysis (SA), principal components analysis (PCA), and mixed linear model (MLM), were widely applied to association analyses [12–14]. In them, the MLM-GWAS procedure has been the most popular procedure, and it has been widely used in *Arabidopsis*, rice, maize, sorghum, and cotton [1, 7, 15–18]. The statisticians further concluded that the above-mentioned GWAS methods (SA, PCA and MLM) based on a whole-genome scan which tests a marker each time were classified as a single-locus model [19, 20]. However, due to a very strict selection criteria of Bonferroni correction, some significant loci associated with the objective traits often were not detected in the single-locus GWAS models [21]. Moreover, the traditional GWAS procedures based on single-locus models, have been chiefly paid close attention to exploring a handful of major QTL in plants, and have been difficult to dissect the full-genome QTL alleles [19, 20]. Nevertheless, it is necessary for molecular breeding to identify genome-wide QTL-allele composition in germplasm resources.

Fortuitously, the multi-locus GWAS (ML-GWAS) models make it possible to explore full-genome QTL alleles. Recently, statisticians have developed a novel restricted two-stage, multi-locus, mutli-allele GWAS (RTM-GWAS) procedure [20] to uncover whole-genome QTL alleles controlling target traits in plants. The RTM-GWAS procedure had been applied to detect a comparatively full-genome QTL-allele system of seed isoflavone content and 100-seed weight in soybean [19, 20, 22]. However, research to detect whole-genome QTL alleles of cotton breeding objective traits was scarce through the RTM-GWAS procedure.

Therefore, to explore the whole-genome QTL alleles significantly associated with cotton yield-related traits, association analyses for three cotton yield-related traits were performed in different planting environments through the RTM-GWAS procedure. To attain this, we used a natural population consisting of 315 upland cotton accessions and developed 13,391 high-quality single nucleotide polymorphisms (SNPs) organized into 9244 SNP linkage disequilibrium blocks (SNPLDBs). In this study, the significant SNPLDBs and the stable QTL associated with three yield-related traits were identified, and the QTL-allele matrices characterizing the population diversity were established via the RTM-GWAS method; and the potential candidate genes were predicted by two RNA-seq data. The results not only provide important insights into the genetic architecture controlling fiber yield traits, but also facilitate breeding high-yielding cotton varieties.

## Results

### Phenotypic variations of three yield-related traits among accessions

**Fig. 1:**
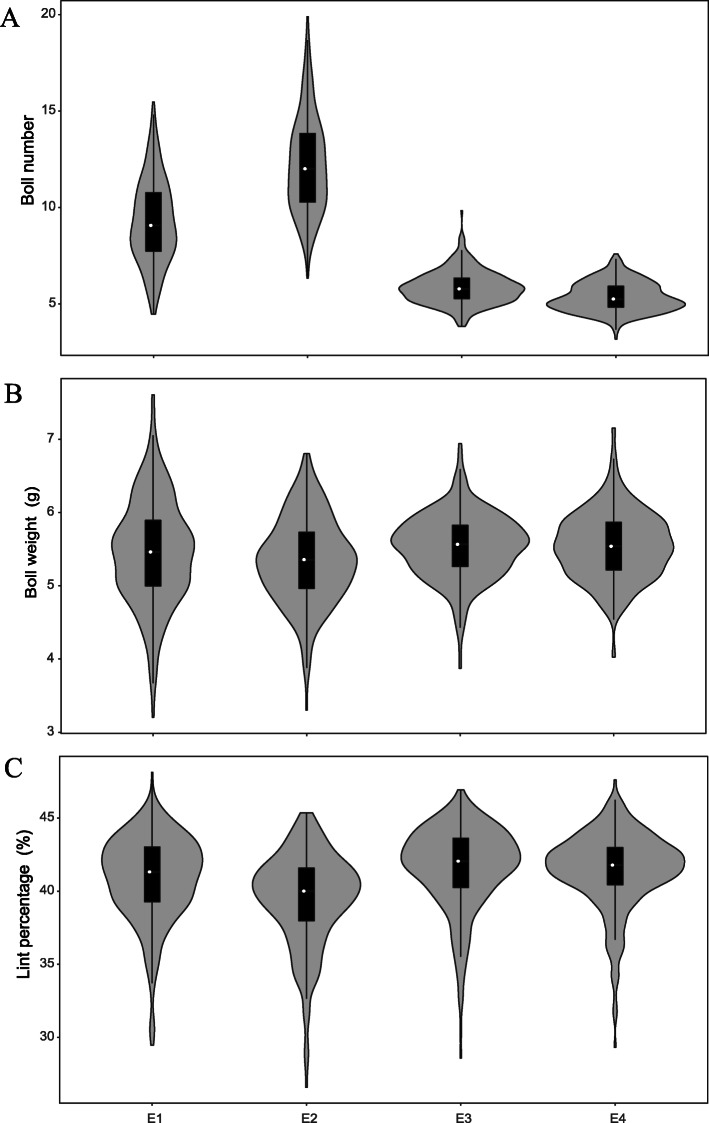
Phenotypic distributions of three yield-related traits among 315 upland cotton accessions in four planting environments (E1-E4). **a** Boll number; **b** boll weight; **c** lint percentage

Three yield-related traits (BN, BW and LP) of the natural population were evaluated in four planting environments (E1–4) during 2014 and 2015. We observed variation range of the average values across four environments for three yield-related traits. The BN, BW, and LP varied from 4.45 to 13.19, 3.60 to 7.13 g, and 28.49 to 47.01%, with an average of 8.18, 5.47 g, and 40.89%, respectively (Fig. 1; Table S1). We observed that the BNs in Anyang (E1 and E2) were strikingly greater than those in Shihezi (E3 and E4). Additionally, positive correlations were also observed between three yield-related traits, however, significant correlation was not found among them (Table S2). The continuous and wide phenotypic distributions suggested that they were segregating characteristic of quantitative trait and were fit for GWAS.

Although these traits exhibited large phenotypic variation, the coefficient of variation (CV) of LP was only comparatively consistent among the four environments. The CV values of the BN and BW in E1–2 were larger than those in E3–4 (Table S1). Analysis of variance (ANOVA) showed that there were highly significant differences among the accessions, environments, and accession-by-environment interactions for the three traits (Table S3). Additionally, the broad-sense heritability (BSH) of the BN, BW and LP was 17.76, 40.80 and 66.67%, respectively (Table S3), revealing that LP and BW were relatively stable inherited, whereas BN trait was greatly affected by the planting-environment factors. These results indicated that the three yield-related traits were significantly influenced by the planting environments.

### The SNPLDB-marker construction and population structure

The association panel consisting of the 315 upland cotton accessions was sequenced through a specific-locus amplified fragment sequencing (SLAF-seq) method. The sequence reads then were aligned against the new upland cotton TM-1 reference genome v2.1 [23] and 1,236,418 SNP markers were developed. With a control criterion of a call rate > 0.90 and a minor allele frequency (MAF) > 0.05, a total of 13,391 high-quality SNPs was retained among the accessions. The SNPs could be located on all 26 chromosomes of upland cotton genome, with 8491 and 4900 SNPs in the At and Dt subgenomes, respectively, and were organized into 9244 SNPLDBs. Based on the SNPLDBs, the association panel was divided into two groups by the PCA and the hierarchical phylogenetic tree, and the linkage disequilibrium (LD) decay distance of the approximate 500 kb was estimated in the population. The detailed results on the PCA and LD of the association panel will be reported in another study (about to be published).

### Detection of QTL alleles through the RTM-GWAS procedure

**Fig. 2:**
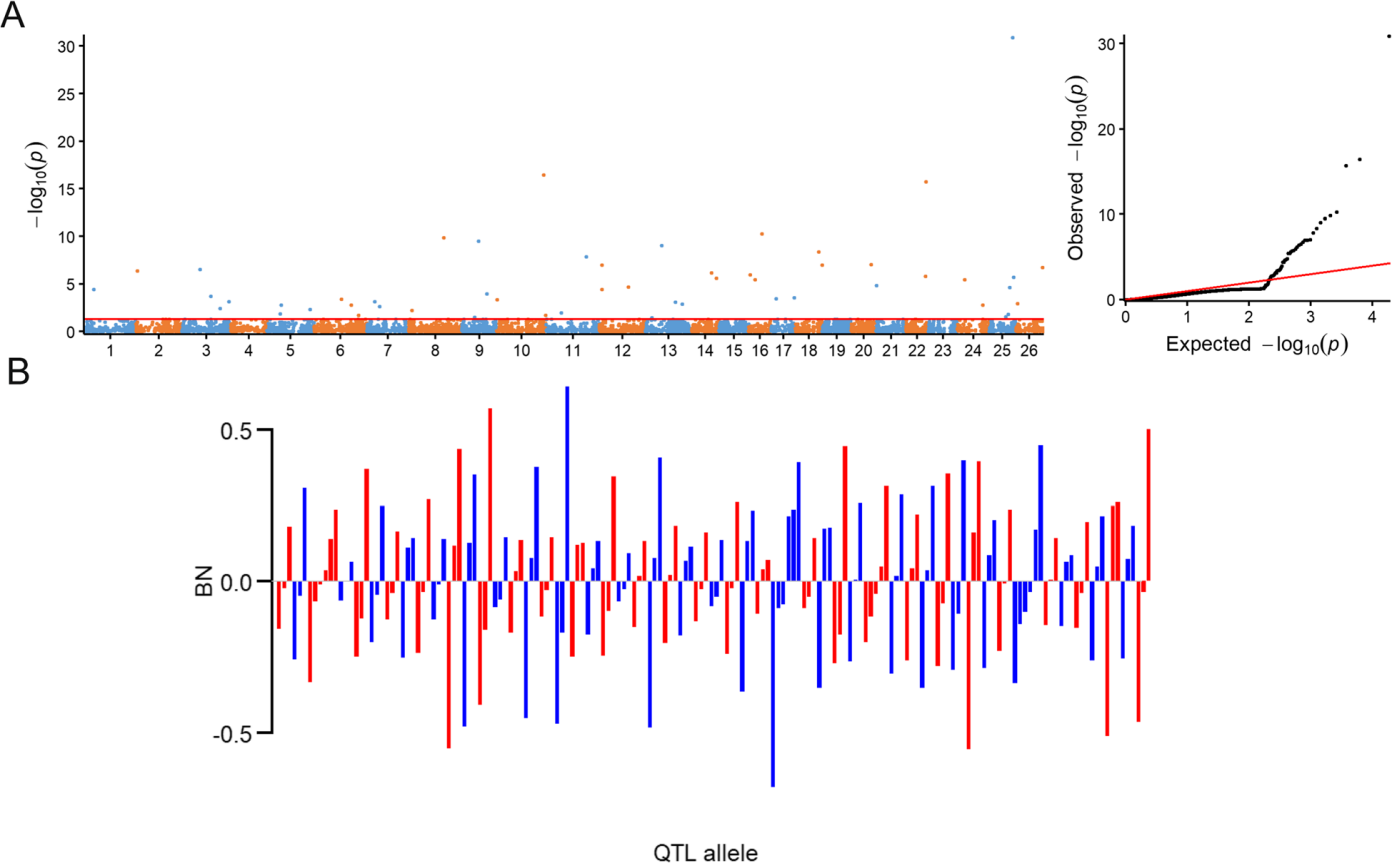
The RTM-GWAS results of BN. **a** Manhattan (left) and quantile–quantile plots (right) of BN. Each dot indicates one SNPLDB marker. The horizontal dashed red line represents the normal significance threshold of 0.05. **b** Distribution of allele effect of the significant SNPLDB loci for BN. The bars above the abscissas mean positive effect values improving fiber quality, while the bars below the abscissas represent negative effect values reducing fiber quality

To detect whole-genome QTL alleles underlying three cotton yield-related traits, the RTM-GWAS procedure based on multi-locus model was applied in the study. Because of the significant difference between the two sites for the BN trait, the RTM-GWAS procedures for multiple environments and single environment were respectively performed in this study. The significant SNPLDBs which could be simultaneously detected in multiple environments and two or more single environments, were thought of as the stable SNPLDB loci. By utilizing the RTM-GWAS procedure of the multiple environments, we identified respectively 53, 70 and 68 significant SNPLDB loci associated with BN, BW and LP (*P* < 0.05; Fig. 2; Table S4).

In the significant SNPLDBs of multiple environments for BN, one stable SNPLDB locus with a high −log_10_(*P*) value and phenotypic variation explanation was not found in the associations, although the 8 SNPLDBs were presented in a single planting environment (Fig. 2a; Table 1). Among the 53 significant loci associated with BN, there were 40 single SNPLDB loci and 13 multiple SNPLDB loci. Based on the stepwise regression analysis, we identified 170 alleles containing 84 positive and 86 negative alleles in these SNPLDB loci. The positive-allele effects ranged from 0.0039 to 0.64, and the negative-allele effects varied from − 0.00034 to − 0.68 (Fig. 2b). In addition, the allele effects of the significant SNPLDB loci could be further organized into a 53 × 315 (locus × accession) matrix, which in truth displayed the genetic variation constitution of the BN trait in the 315 upland cotton accessions (Figure S1A).

**Table 1:** The significant SNPLDB loci associated with three yield-related traits in upland cotton

Traits	Locus	Chr.	Position	-log_10_(*P*)	PVE(%)	Common environments ^a^
BN	LDB_10_108252822	A10	108,252,822	16.46	0.58	E2(7.68)
	LDB_8_80957905_80958114	A08	80,957,905	9.87	0.97	E2(3.30)
	LDB_9_38930641	A09	38,930,641	9.52	0.51	E2(2.58)
	LDB_18_53807715	D05	53,807,715	8.38	0.37	E3(2.32)
	LDB_3_42046528_42046540	A03	42,046,528	6.52		E2(4.92)
	LDB_16_4143574	D03	4,143,574	5.94	0.38	E2(4.39)
	LDB_5_32745305	A05	32,745,305	2.80		E1(3.07)
	LDB_5_30624560	A05	30,624,560	1.84	0.22	E2(5.03)
BW	LDB_25_61293136^b^	D12	61,293,136	28.02	1.05	E1(5.75), E2(7.39), E4(9.79)
	LDB_7_11434307	A07	11,434,307	19.97	3.12	E3(13.44)
	LDB_18_60784,945	D05	60,784,945	19.01	2.68	E1(4.96)
	LDB_8_117999939	A08	117,999,939	18.24	0.97	E3(9.25)
	LDB_24_62501704_62501749	D11	62,501,704	14.84	0.56	E1(6.03)
	LDB_18_62137879	D05	62,137,879	14.07	1.31	E4(10.43)
	LDB_1_27714781	A01	27,714,781	13.53	2.33	E1(1.71)
	LDB_23_7162905	D10	7,162,905	12.59	0.58	E4(3.13)
	LDB_3_101231660	A03	101,231,660	8.52	0.20	E3(11.57)
	LDB_5_2524535	A05	2,524,535	7.65	1.00	E1(1.69)
	LDB_15_21962239	D02	21,962,239	2.02		E4(2.61)
	LDB_13_31802375	A13	31,802,375	1.31		E1(8.65)
LP	LDB_2_98957055_98957100^b^	A02	98,957,055	46.97	0.98	E3(21.27), E4(17.27)
	LDB_20_52488458^b^	D07	52,488,458	40.68	5.09	E2(10.18), E3(12.10)
	LDB_6_81838215_81856977	A06	81,838,215	39.14	3.67	E4(13.50)
	LDB_19_62926589^b^	D06	62,926,589	29.91	2.60	E2(14.57), E3(4.25),
	LDB_15_33683466_33683486^b^	D02	33,683,466	22.99	0.19	E3(8.76), E4(8.64)
	LDB_6_87041656_87041922^b^	A06	87,041,656	21.47	2.01	E1(4.66), E2(3.48)
	LDB_22_5519816_5520088	D09	5,519,816	17.17	2.12	E3(10.04)
	LDB_5_6292927	A05	6,292,927	16.95	1.20	E4(17.58)
	LDB_13_14883849_14884035	A13	14,883,849	6.83	0.35	E2(7.38)
	LDB_15_6072794	D02	6,072,794	6.01	0.77	E3(2.25)
	LDB_15_58321721	D02	58,321,721	3.90		E1(1.66)
	LDB_12_92891416_92891433	A12	92,891,416	1.61	0.08	E1(2.93)

^a^ Figures in brackets denote -log_10_(*P*) values in the planting environments. ^b^ labels the six stable SNPLDB loci

*BN* Boll number, *BW* Boll weight, *LP* Lint percentage, *Chr.* Chromosome, *PVE* Phenotypic variation explanation

**Fig. 3:**
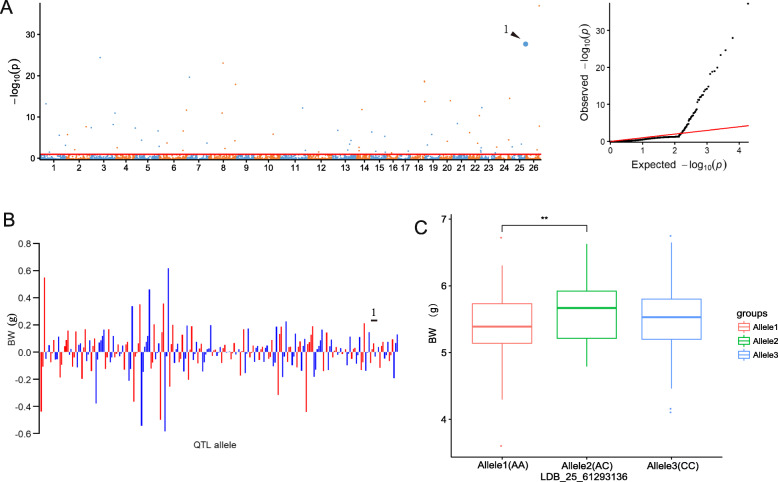
The RTM-GWAS results of BW. **a** Manhattan (left) and quantile–quantile plots (right) of BW. **b** Distribution of allele effect of the significant SNPLDB loci for BW. The number 1 indicates the significant SNPLDB locus LDB_25_61293136. **c** The BW differences of the stable SNPLDB locus LDB_25_61293136 among different alleles. Allele1–3 represent AA, AC and CC, respectively. * and ** indicate respectively 5% and 1% significance level

In the significant SNPLDBs of multiple environments for BW, one stable SNPLDB locus (LDB_25_61293136) was detected in three single planting environments, and 11 SNPLDBs were also presented in a single planting environment (Fig. 3a; Table 1). The stable SNPLDB locus LDB_25_61293136 located on chromosome 25 (D12) were simultaneously identified in the three single planting environments (E1, E2 and E4), with the higher −log_10_(*P*) value (28.02) and phenotypic variation explanation (1.05%). Among the 70 SNPLDB loci associated with BW, 15 loci of them included multiple SNPs, whereas the remaining 55 loci contained single SNP. The allele-effect values ranged from 0.0011 to 0.62 g with an average of 0.47 g for the 115 positive alleles, and varied from − 0.000070 to − 0.58 g with a mean of − 0.10 g for the 113 negative alleles (Fig. 3b). The stable SNPLDB locus LDB_25_61293136 had three allele types including AA, AC and CC with effect value of − 0.12, 0.045 and 0.073, respectively (Fig. 3b); and the BW values of the accessions with the negative allele (AA) were significantly lower than those with the heterozygote allele (AC), whereas was not significantly lower than those with the positive allele (CC) (Fig. 3c). Additionally, for the 70 significant SNPLDB loci with 147 haplotypes/alleles, the allele effects were organized into a 70 × 315 (locus × accession) matrix, containing the core genetic information for the improvement of the BW trait in the association panel (Figure S1B).

**Fig. 4:**
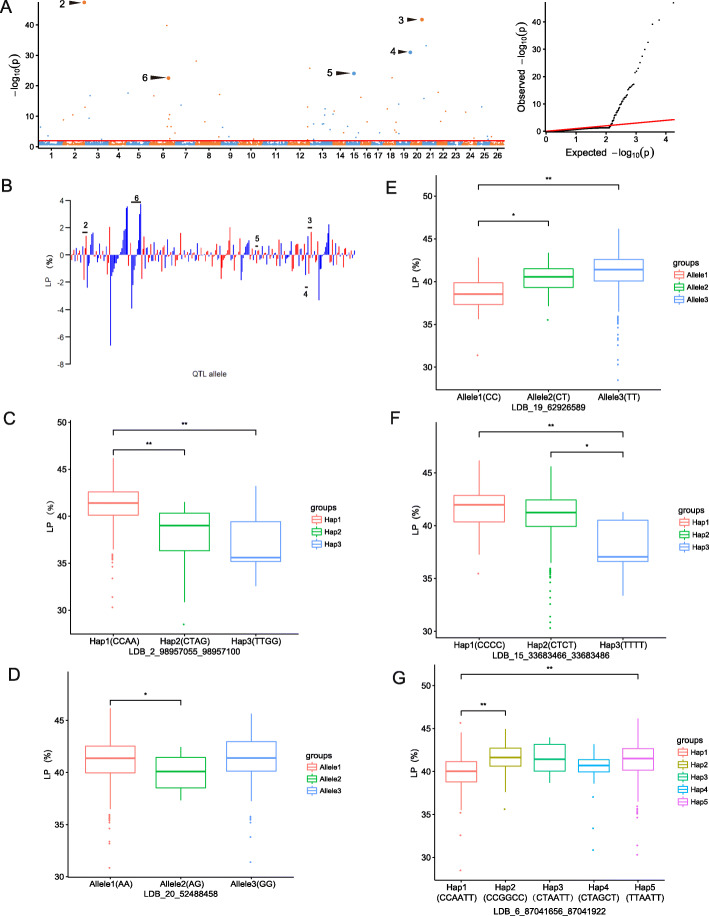
The RTM-GWAS results and haplotype analysis of LP. **a** Manhattan (left) and quantile–quantile plots (right) of LP. **b** Distribution of allele effect of the significant SNPLDB loci for LP. The number 2–6 indicate respectively the five significant SNPLDB loci LDB_2_98957055_98957100, LDB_20_52488458, LDB_19_62926589, LDB_15_33683466_33683486, and LDB_6_87041656_87041922. **c**-**g** The LP differences of the five stable SNPLDB loci among different haplotypes/alleles. **c** LDB_2_98,957,055_98957100, Hap1(CCAA), Hap2(CTAG) and Hap3(TTGG); **d** LDB_20_52488458, Allele1–3 represent AA, AG and GG, respectively; **e** LDB_19_62926589, Allele1–3 represent CC, CT and TT, respectively. **f** LDB_15_33683466_33683486, Hap1(CCCC), Hap2 (CTCT), Hap3(TTTT); **g** LDB_6_87041656_87041922, Hap1(CCAATT), Hap2 (CCGGCC), Hap3(CTAATT), Hap4(CTAGCT) and Hap5(TTAATT)

In the significant SNPLDBs associated with LP in multiple environments, five stable SNPLDB loci were detected in two single planting environments, and seven SNPLDBs were also observed in a single planting environment (Fig. 4a; Table 1). Among the five stable SNPLDB loci, the peak SNPLDB locus LDB_2_98957055_98957100 was positioned on chromosome 2 (A02) with the highest −log_10_(*P*) value (46.97) and phenotypic variation explanation (3.98%), which was simultaneously identified in the two single planting environments (E3 and E4); and the second highest locus LDB_20_52488458 was distributed in chromosome 20 (D07), which had a − log_10_(*P*) value of 40.68 and explained the largest phenotypic variation of 5.09%. Among the 68 associated loci with LP, 23 of them were multiple-SNP loci, and the rest of them were single-SNP loci. The allele-effect values ranged from 0.0034 to 3.73% with an average of 0.67% for the 124 positive alleles, and varied from − 0.012 to − 6.65% with a mean of − 0.64% for the 131 negative alleles (Fig. 4b). For example, the peak SNPLDB locus LDB_2_98957055_98957100 had three haplotypes including Hap1, Hap2 and Hap3 with an effect value of 0.47, − 1.86, and − 1.39%, respectively (Fig. 4b); the accessions carrying the Hap1(CCAA) exhibited a significantly increased LP, compared with the accessions carrying the Hap2(CTAG) and Hap3(TTGG) (Fig. 4c). Within another association locus LDB_20_52488458, there were three alleles including AA, AG and GG with effect of − 0.26, − 1.40, and 1.66%, respectively (Fig. 4b); while the significant phenotypic difference was not observed between the lines containing the AA and GG allele (Fig. 4d). Another significant signal LDB_6_87041656_87041922 had five haplotypes including Hap1(CCAATT), Hap2 (CCGGCC), Hap3(CTAATT), Hap4(CTAGCT) and Hap5(TTAATT), and the LP values of the accessions containing the Hap2 and Hap5 were significantly higher than those containing the Hap1 (Fig. 4g). For the other two stable SNPLDB loci (LDB_19_62926589 and LDB_15_33683466_33683486), the LP values of the accessions with the favorable haplotypes/alleles were significantly higher than those with the unfavorable haplotypes/alleles (Fig. 4e, f). Moreover, the effects of the 68 significant LP SNPLDB loci containing 156 haplotypes/alleles could be established into a 68 × 315 (locus × accession) matrix for the whole genetic variation information of the LP trait (Figure S1C).

**Fig. 5:**
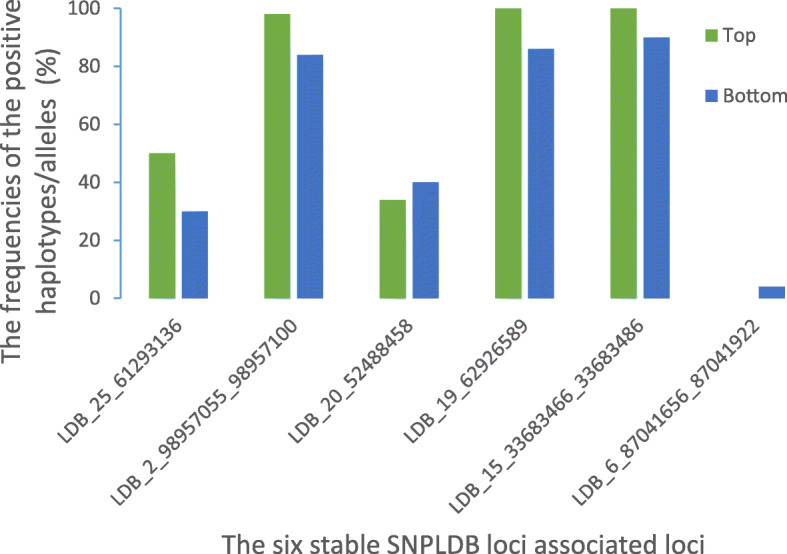
The frequencies of positive haplotypes/alleles for the six stable SNPLDB loci in the top 50 lowest and the bottom 50 highest yield accessions

From the QTL-allele matrices of three target traits, we found the regularity that the frequencies of the positive haplotypes/alleles in the superior accessions were more than those in the inferior ones. The phenomenon could be intuitively observed between the highest and lowest yield accessions. Take LP trait as an example, the cumulative number of the positive haplotypes/alleles in the 50 highest lines (1943 with a mean of 38.86 per line) was larger than that in the 50 lowest LP ones (1818 with an average of 36.36 per line). Correspondingly, the cumulative number of the negative haplotypes/alleles in the 50 highest accessions (1796 with an average of 35.92 per accession) was less than that in the 50 lowest LP ones (1998 with a mean of 39.96 per accession). Despite of phenotypic differences among the 315 accessions, any one of them contained simultaneously the positive and negative alleles. For instance, a fine cotton variety “lumianyan28”, the number of positive alleles of the BN, BW and LP was 34, 44 and 44, respectively; and the number of negative alleles was 32, 42 and 42, respectively. In addition, we also gave attention to the significant SNPLDB loci. For the stable BW SNPLDB locus LDB_25_61293136, the frequency of the positive alleles in the top BW 50 lines was obviously higher than that in the bottom 50 ones. For the five stable SNPLDB loci for LP, three (LDB_2_98957055_98957100, LDB_19_62926589, and LDB_15_33683466_33683486) of them conformed to the law that the frequencies of the positive haplotypes/alleles in the top LP 50 lines were clearly higher than that in the bottom 50 ones (Fig. 5).

In a brief summary of the QTL-allele detection, our results were to (1) a total of 53, 70 and 68 significant SNPLDB loci associated with BN, BW and LP were respectively identified through the multiple-environment RTM-GWAS procedure, and the effects of the significant SNPLDB loci were organized into the QTL-allele matrices of three target traits; (2) one and five steadily associated SNPLDB loci with BW and LP were respectively detected; (3) the QTL alleles with extremely high or low effects were not necessarily the ones from the peak SNPLDB loci with the largest –log_10_(*P*) value and phenotypic variation explanation; (4) for the significantly associated loci, the frequencies of the positive alleles in the superior accessions were generally more than those in the inferior ones, but not all the loci completely conformed to the underlying laws. The results offered important insights into the genetic basis underlying yield-related traits in upland cotton.

### Identification and prediction of potential candidate genes

Based on the above results, the six stable SNPLDB loci associated with BW and LP were identified via the RTM-GWAS procedure, and they should be major-effect QTL controlling cotton yield-related traits. Therefore, we mainly focused on the six stable SNPLDB loci. By reference to the LD decay distances of SNPLDBs of the association panel and the SNP-linked fragment in the most of previous cotton studies (200 kb) [4, 24–26], the six genome fragments (±200 kb around the six stable SNPLDB loci) were recommended as target scopes of candidate genes. According to the upland cotton TM-1 reference genome v2.1 [23], a total of 115 genes were annotated in the six target genome fragments (Table S5). Through aligning the genomic locations of the genes, we discovered that the stable SNPLDB locus (LDB_15_33683466_33683486) was located within the coding sequence (CDS) of the gene (*GH_D02G1202*). Also, we found that the SNPLDB locus LDB_19_62926589 was positioned in the promoter region of candidate gene *GH_D06G2161*.

**Fig. 6:**
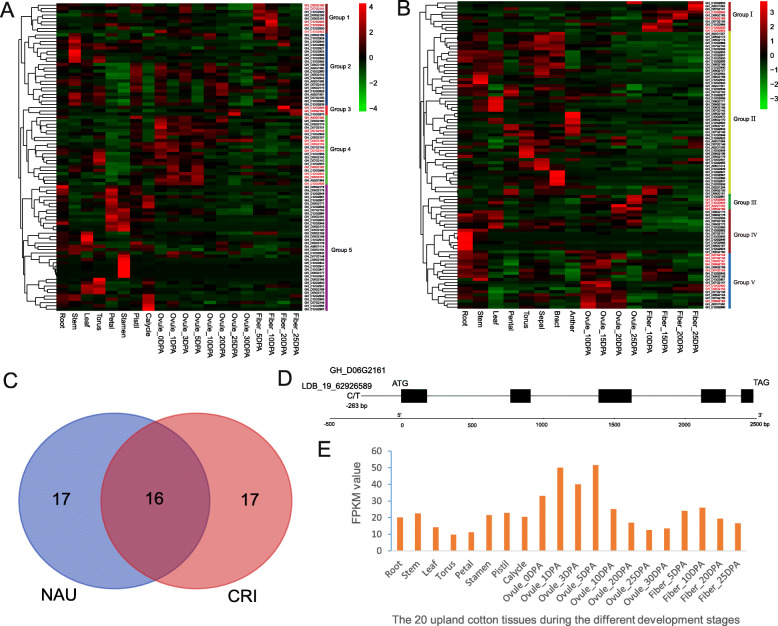
Identification of the potential candidate genes associated with BW or LP. **a** Heatmap of expression patterns of the 93 putative candidate genes among 20 upland cotton (TM-1) tissues by the RNA-seq data from Nanjing Agricultural University (NAU). Red indicates high expression, and green indicates low expression. **b** Heatmap of expression patterns of the 100 putative candidate genes among 16 upland cotton (TM-1) tissues by the RNA-Seq data from Institute of Cotton Research of Chinese Academy of Agricultural Sciences (CRI), the16 genes were highlight in red in the **a** and **b**. **c** Venny diagram of the common genes between two RNA-seq data (NAU and CRI). **d** Gene structure of *GH_D06G2161* and the position of the SNPLDB locus LDB_19_62926589 in the gene promoter region. E. Transcriptomic level of *GH_D06G2161* in 20 different tissues based on the fragments per kilobase of transcript per million mapped reads (FPKM) values

On the basis of the normalized fragments per kilobase of transcript per million mapped reads (FPKM) values of the genes from the Nanjing Agricultural University (NAU) RNA-seq [27], 93 of the 115 genes were highly expressed in at least one of 20 upland cotton tissues, and were divided into five different groups (Group 1–5). Among the five groups, Group 1 including 9 genes was mainly expressed in fibers of 5 and 10 days post anthesis (DPA), Group 2 including 22 genes was principally expressed in stem and floral organs, Group 3 contained only three genes was mainly expressed in fibers of 20 and 25 DPA, Group 4 contained 21 genes was mainly expressed during the five ovule-development stages, and Group 5 contained 38 genes was mainly expressed in floral organs (Fig. 6a). Additionally, based on the other FPKM values of the genes from Institute of Cotton Research of Chinese Academy of Agricultural Sciences (CRI) RNA-seq, 100 of the 115 genes were highly expressed in at least one of 16 upland cotton tissues, and could also be divided into five different groups (Group I-V). Among the five groups, 10 genes were assigned to Group I that were highly expressed in the fibrous tissues; 53 genes were assigned to Group II that were highly expressed in the stem, leaf and floral organs, 5 genes belonged to Group III that were highly expressed in ovule of 20 or 25 DPA, 14 genes belonged to Group IV that were highly expressed in root, and the remaining 18 genes belonged to Group V that were mainly expressed during the five ovule-development stages (Fig. 6b). Both sets of genes (33 each) expressed mainly in fibers and ovules were respectively identified via the NAU and CRI RNA-seq (Table S6); and 16 of them were in common between two RNA-seq data and should be potential candidate genes for cotton yield-related traits (Fig. 6c; Table 2). In addition, the 16 genes were respectively assigned to Group- 1,3, 4 and Group- I, III, V (Fig. 6c). Therefore, we speculated that the genes assigned to the Group- 1,3, 4 and Group- I, III, V might be closely related to the target traits.

**Table 2:** The 16 potential candidate genes controlling target traits predicted by two RNA-seq data

Gene ID	Gene Name	Description	Chr.	Start	End	Length (bp)
*GH_D06G2180*	*COX5B-2*	Cytochrome c oxidase subunit 5b-2, mitochondrial	D06	63,060,142	63,062,960	2819
*GH_D07G2154*	*Acot9*	Acyl-coenzyme A thioesterase 9, mitochondrial	D07	52,623,091	52,626,062	2972
*GH_D12G2925*			D12	61,121,419	61,121,910	492
*GH_D12G2954*	*bro1*	Vacuolar protein-sorting protein bro1	D12	61,342,250	61,347,159	4910
*GH_D12G2952*	*HDG11*	Homeobox-leucine zipper protein HDG11	D12	61,327,801	61,332,967	5167
*GH_D12G2950*	*MYB52*	Transcription factor MYB52	D12	61,304,801	61,306,952	2152
*GH_D06G2164*	*At1g12280*	Probable disease resistance protein At1g12280	D06	62,950,810	62,953,515	2706
*GH_A02G1555*	*ZHD1*	Zinc-finger homeodomain protein 1	A02	98,782,762	98,783,781	1020
*GH_D07G2145*			D07	52,419,774	52,428,479	8706
*GH_D06G2165*	*ROD1*	Phosphatidylcholine:diacylglycerol Cholinephosphotransferase 1	D06	62,958,995	62,961,025	2031
*GH_D06G2153*	*NDC80*	Probable kinetochore protein NDC80	D06	62,840,437	62,844,537	4101
*GH_D07G2144*	*PYRAB13050*	UPF0056 membrane protein PYRAB13050	D07	52,395,029	52,395,241	213
*GH_D06G2162*	*KEG*	E3 ubiquitin-protein ligase KEG	D06	62,930,295	62,932,383	2089
*GH_D12G2934*	*xpr1*	Xenotropic and polytropic retrovirus receptor 1 homolog	D12	61,194,759	61,195,031	273
*GH_D06G2161*	*asnS*	Asparagine--tRNA ligase	D06	62,926,856	62,929,336	2481
*GH_D12G2926*	*rpc11*	DNA-directed RNA polymerase III subunit RPC10	D12	61,125,552	61,126,483	932

For the 16 potential candidate genes, 14 of them had some biological function annotations. Seven genes encode the binding proteins related to membrane, zinc finger and disease resistance, etc.; six genes encode enzymes, such as oxidase, thioesterase, diacylglycerol cholinephosphotransferase and E3 ubiquitin-protein ligase, asparagine-tRNA ligase, etc.; one of the remaining genes encodes MYB transcription factor (Table 2). Furthermore, we performed function prediction for the 16 genes by Gene Ontology (GO) and Kyoto Encyclopedia of Genes and Genomes (KEGG) pathway items. The GO results showed that four genes (*GH_D12G2952*, *GH_D12G2950*, *GH_A02G1555* and *GH_D12G2926*) were related to a molecular function by which a gene product interacts selectively and non-covalently with DNA (GO:0003677); and two genes (*GH_D06G2164* and *GH_D06G2161*) involved in the process of interacting selectively and non-covalently with any protein or protein complex (GO:0005515) (Table S7). By the KEGG pathway analysis, 15 pathways were predicted in two genes (*GH_D06G2180* and *GH_D12G2926*) and the common metabolic pathway (ko01100) of them was found (Table S8). More importantly, the gene *GH_D06G2161* belonged to the class-II aminoacyl-tRNA synthetase family which are ubiquitous and evolutionarily conserved enzymes that catalyze the highly specific acylation of amino acids to cognate tRNAs, which was expressed mainly in the early ovule-development stages, and the SNPLDB locus LDB_19_62926589 was detected in its promoter region (Fig. 6d, e). Gene homology analysis showed that *GH_D06G2161* is a homologue of *asnS* gene involved in asparagine-tRNA ligase activity in *Mycoplasma arthritidis* [28]. The *asnS* gene (*AT5G32440*) is a ubiquitin system component cue protein mRNA in *Arabidopsis thaliana* [29]. However, the function of these genes is still uncertain.

## Discussion

### Phenotypic trait difference

At present, the top five cotton-producing countries are India, the USA, China, Pakistan, and Brazil; they together produce more than 70% of the world’s cotton in 2019. China ranks in fourth in terms of area harvested, while it produces a secondary world’s cotton yield. Even so, China is still the largest country of cotton fiber imports and domestic consumption (USDA-FAS 2019) [30]. In addition, cotton harvested area has been falling for at least 5 years in China, so improving fiber yield is still a major goal in Chinese cotton breeding practice. In the present study, three cotton yield-related traits (BN, BW and LP) were evaluated in the four planting environments. We found that the phenotypic measured values of the three yield-related traits exhibited a high-degree of diversity. The phenotypic investigation of the study was significant for offering some very fine germplasms in future cotton yield breeding programs. Moreover, three cotton yield-related traits, especially the BN trait, were significantly influenced by the planting environments, and the phenotypic measured values in the E3–4 environments were highly significantly lower than those in the E1–2. That is because plant density might be one of the most important factors impacting the BN per plant, besides climatic factors of light, temperature, and water. In the field experiment, plant density of the E1–2 environments (a plant per 0.18 m^2^) was obviously lower than that of the E3–4 environments (a plant per 0.043 m^2^). A higher plant density generally increases intra-plant competition for resources, such as light, water and nutrients, and reduces cotton fiber yield per plant [31, 32]. For an individual cotton plant, therefore, its fiber yield might be greatly affected by plant density. In the study, the BN investigation values were influenced by the plant density difference between the two experimental sites.

### The comparison of the GWAS results

Cotton fiber yield is one of the most important traits and is controlled by quantitative trait genes. Quantitative traits are often greatly affected by environment. Hence, most QTL could be detected only in individual environment, while only a handful of stable QTL could be simultaneously detected in several planting environments. The previous study thought of the QTL detected simultaneously in multiple environments as stable QTL [33]. In the study, the significant SNPLDBs which could be simultaneously detected by the RTM-GWAS procedures for multiple environments and two or more single environments, were considered of as stable QTL for the objective traits. By utilizing the comprehensive analysis of the multiple environments and single environment, the six stable SNPLDB loci associated with BW or LP were detected through the RTM-GWAS procedures.

Previously, some SNP loci associated with yield-related traits have been detected via the GWAS method in upland cotton [1, 4, 6–8], and the GWAS results are listed in Table S9. According to recent research, some stable BN-QTL (D02:55.00–58.20 Mb, A02:79.10–79.45 Mb, and D08:2.80–3.10 Mb), BW-QTL (D09:47.70–47.75 Mb and A02:74.94–75.56 Mb), and LP-QTL (A02:74.71–75.56 Mb, A02:79.10–79.45 Mb, A06:102.50–103.60 Mb, A08:52.09–52.10 Mb, D02:1.30–1.60 Mb, D02: 2.20–2.36 Mb, D03:30.00–40.00 Mb, D08:2.80–3.10 Mb, and D12:55.00–58.20 Mb) had been identified in the last 5 years [1, 4, 6–8]. In the previous investigations, the QTL associated with the three yield-related traits were explored by utilizing MLM-GWAS based on single-locus model, and most of them were primarily positioned on chromosomes A02, D02, and D08. In this study, one stable BW-SNPLDB locus LDB_25_61293136 (D12) and five stable LP-SNPLDB loci including LDB_2_98957055_98957100 (A02), LDB_20_52488458 (D07), LDB_19_62926589 (D06), LDB_15_33683466_33683486 (D02) and LDB_6_87041656_87041922 (A06), were identified via the RTM-GWAS procedure. To verify the authenticity and novelty of the significant SNPLDB loci associated with target traits, the stable QTL identified in the study were compared with those of the previous GWAS results. The results showed that the five stable SNPLDB loci except the locus LDB_2_98,957,055_98957100 may be a novel QTL for BW or LP trait. Moreover, the 16 potential candidate genes which were specially and highly expressed in ovules or fibers were forecasted in the neighbor genome fragment of the stable SNPLDBs by the two RNA-seq data. We speculated that the genes might be help regulate growth and development of cotton ovules or fibers and were closely related to lint yield.

### The superiority of the RTM-GWAS procedure

In plant molecular breeding process, it is very important to detect whole-genome QTL in breeding resources by GWAS, but the previous single-locus GWAS procedures have been mainly concentrated on identifying a spot of major QTL alleles [19, 20]. Our purpose of study on the genetic basis for breeding objective traits was not only to detect a few major QTL, but also to identify the full QTL-allele system in a cotton germplasm population via the improved GWAS procedure. Hence, to reveal the whole-genome QTL controlling three cotton yield-related traits, some GWAS procedures with high detection efficiency and power should be applied in our study. Recent studies have proved that the RTM-GWAS program has the higher QTL detection efficiency and strength than the other GWAS models including MLM in soybean [19, 20, 22, 34]. Due to the apparent advantages of the RTM-GWAS procedure in the whole QTL alleles, it was used for detecting yield-related QTL alleles in our study. Compared with the previous single-locus GWAS methods, more significant QTL associated with cotton yield-related traits were detected via the RTM-GWAS procedure. In this study, a total of 68 significant SNPLDB loci associated with LP was identified through the multiple-environment RTM-GWAS procedure, whereas only 12 significant SNP loci for LP were detected via the MLM-GWAS method in our previous report [6]. Similarly, the stable LP-QTL detected through the RTM-GWAS procedure (5) were more than those through the MLM-GWAS (2). The comparison result showed that the RTM-GWAS method had advantage over the MLM-GWAS in the dissection of more QTL for objective traits.

In addition, the RTM-GWAS procedure can also provide the QTL-allele matrix with the comparatively overall genetic information including the significant SNPLDB loci and their allele effects, population allele constitution, and frequency distribution of each locus. In the study, the QTL-allele matrices of three yield-related traits (BN, BW and LP) were established by utilizing the positive and negative alleles of the significant SNPLDB loci. We achieved result that the superior accessions had more positive haplotypes/alleles than those in the inferior ones from the QTL-allele matrices. The phenomenon could be directly observed between the top and bottom phenotypic value accessions. However, the QTL-allele matrix could be not directly organized in the traditional GAWS models.

## Conclusions

A total of 53, 70 and 68 significant SNPLDB loci associated with BN, BW and LP were respectively detected in the panel composed of 315 upland cotton accessions through the multiple-environment RTM-GWAS program. The haplotype/allele effects of the significant SNPLDB loci were calculated and the QTL-allele matrices were organized for offering the abbreviated genetic composition of the natural population. Among the significant SNPLDB loci of multiple environments, six of them were simultaneously identified in two or more single planting environments and were thought of as the stable SNPLDB loci. Therefore, the six stable SNPLDB loci were focused and a total of 115 genes were annotated in their nearby regions, and 16 potential candidate genes controlling target traits of them were predicted by two RNA-seq data. One of 16 genes (*GH_D06G2161*) was mainly expressed in the early ovule-development stages, and the SNPLDB locus LDB_19_62926589 was mapped in its promoter region. The study identified the QTL alleles and candidate genes that could provide important insights into the genetic basis of yield-related traits in upland cotton.

## Methods

### Plant materials and growth conditions

An association panel consisted of 290 lines collected from China, 21 accessions introduced from the United States of America (USA), and 4 accessions from the former Soviet Union. Seeds of the Chinese cotton varieties were obtained from our germplasm collection, and seeds of the rest of germplasm lines were collected from germplasm bank of Institute of Cotton Research of Chinese Academy of Agricultural Sciences (Table S10). All the upland cotton accessions were planted in four natural environments including E1 and E2 [Anyang (36.13°N, 114.80°E) in Henan Province in 2014 and 2015, respectively], and E3 and E4 [Shihezi (44.52°N, 86.02°E) in Xinjiang Production and Construction Group in 2014 and 2015, respectively]. All the accessions were grown in accordance with a randomized complete block design experiment with three replications in each environment. The cultivars were grown in a single-row plot (400 cm in length and 80 cm in width with 20 cm spacing between plants) with about 20 plants in E1 and E2, whereas all the accessions were planted in double-row plots (200 cm in length and 76 cm in width with 10 cm spacing between plants) with approximately 40 plants in E3 and E4. Field management followed routine farming methods.

### Phenotypic trait measurements

Three yield-related traits (BN, BW and LP) were measured in each environment. In early September of each experimental year, the BN trait was surveyed from ten continuous plants in the middle of each row. In October of each experimental year, twenty normally opened bolls from the central part of plants of each cultivar were hand harvested and weighed to reckon the BW trait. Then the fiber samples were carefully and thoroughly peeled off by using a cotton gin, and fiber weight (FW, g) were obtained by electronic balance. Then the LP values each line were calculate by a formula of LP (%) = FW (g)/BW (g) × 100%. For the three yield-related traits, their broad-sense heritability (BSH) were calculated in Generalized Linear Model (GLM) and ANOVA of the phenotypic data was conducted using IBM SPSS 22.0 software.

### SNP calling and SNPLDB assembly

Genomic DNA from all the lines was extracted from the young leaf using a corrected cetyltrimethylammonium bromide (CTAB) approach [35], and sequencing reads were acquired in Illumina HiSeq 2500 system (Illumina, Inc., San Diego, CA, USA) by the SLAF-seq method [36, 37]. Then, we use BWA mem software to align high-quality reads of the 315 accessions to a new upland cotton TM-1 reference genome v2.1 [23], all variant calling pipeline were based on GATK best practise, with a bit modification. Firstly, we use BWA mem software to align reads to reference genome using three default paramters (−M -a -t8), second picard were used to mark duplicate, third we use the GATK Haplotyper-Gvcftyper pipeline to call variants and filtered by hard parameters privade by broad institude (QD < 2.0 || FS > 60.0 || MQ < 40.0 || MQRankSum < − 12.5|| ReadPosRankSum < − 8.0 || SQR > 3.0) [38, 39]. The SNPs were filtered with a criterion of a maximum missing > 0.10 and a minor allele frequency (MAF) < 0.05.

The Haploview software was used to calculate haplotype blocks and posterior probability of multi-marker type for determining the final SNPLDB datasets [40, 41]. The confidence interval method was used to define blocks with default settings in Haploview, except SNP blocks with more than 200 kb genome distance [42]. Like single SNP alleles, the multiple SNP makers within the same one LD block were organized into an SNPLDB with several haplotypes.

### The RTM-GWAS procedure

Based on the GSC matrix estimated from whole-genome SNPLDB markers, a neighbour-joining tree of the 315 upland cotton varieties was constructed using Mega7.0 software. Then, the population structure deviation was corrected by the top ten eigenvalues of the GSC matrix estimated from whole genome SNPLDBs. In the study, we used the RTM-GWAS procedure integrated with population structure reclamation based on an elastic genetic similarity coefficient (GSC) matrix (the elastic GSC matrix and GSC eigenvectors were showed in Table S11), and two-stage association analyses including primary screening of SNPLDBs under the single-locus model and stepwise regression analysis in the multi-locus model. The RTM-GWAS procedure was conducted by a reported reference [20]. Based on the 9244 SNPLDBs with multiple alleles, the RTM-GWAS procedures for multiple environments and four single environments for three yield-related traits (BN, BW and LP) were respectively performed by using phenotypic values across four planting environments. To lower a very strict selection criteria of Bonferroni correction and detect the genome-wide QTL alleles, the SNPLDBs of *P* value < 0.05 (a normal significance level) were thought of as significant loci in two-stage association analyses of the RTM-GWAS procedure, referring to the previous studies in soybean [19, 20].

For the significant SNPLDB loci associated with target traits, the allele-effect values for each locus were reckoned by the second-stage association analysis of the RTM-GWAS procedure [20]. According to the allele-effect values, then, the positive and negative alleles were determined for all the significant SNPLDB loci. Finally, the QTL-allele matrices for objective traits were created through R Version 3.6.0, making use of the data of the calculated allele-effect values of all the significant SNPLDBs via a cross software of the RTM-GWAS procedure as previously described [20].

### Expression analysis of potential candidate genes

A few SNPLDB loci which could be simultaneously detected in two or more planting environments, were considered as stable loci associated with objective traits. For the stable and top SNPLDB loci, the mean phenotypic value of each haplotype/allele was calculated by the phenotypic values over the cotton accessions with each SNPLDB type. The two-tailed T-tests of the phenotypic values of each haplotype/allele for the stable loci were performed by making use of IBM SPSS 22.0 software. Chromosomal positions of the stable trait-associated SNPLDB loci were applied to explore potential candidate genes in the upland cotton TM-1 reference genome v2.1 [23]. According to the region of SNP-linked candidate genes detected in previous studies of cotton [4, 24–26], the screening genomic fragments of the candidate genes for the stable SNPLDB loci were ± 200 kb around the markers in the study.

A set of RNA-seq data from 20 upland cotton (TM-1) tissues (including root, stem, leaf, torus, petal, stamen, pistil, calycle, Ovule_0 DPA, Ovule_1 DPA, Ovule_3 DPA, Ovule_5 DPA, Ovule_10 DPA, Ovule_20 DPA, Ovule_25 DPA, Ovule_30 DPA, fiber-5 DPA, fiber-10 DPA, fiber-20 DPA and fiber-25 DPA) were obtained by the NAU and were available on the NCBI SRA database (SRA: PRJNA248163) [27], and the FPKM values were reckoned to show the gene expression levels. The FPKM values of another set of RNA-seq data from 16 upland cotton (TM-1) tissues (including root, stem, leaf, petal, torus, sepal, bract, anther, Ovule_10 DPA, Ovule_15 DPA, Ovule_20 DPA, Ovule_25 DPA, fiber_5 DPA, fiber_10 DPA, fiber_20 DPA and fiber_25 DPA), were gained by a website (http://grand.cricaas.com.cn/page/tools/expressionVisualization) from the CRI. The heatmaps of the putative candidate gene expression styles and box plots for the phenotypic values were drawn by the software center (https://www.omicshare.com/tools/home/index/index.html). For all the potential candidate genes, the GO enrichment analysis and KEGG analysis were performed using the cotton biological information website (https://cottonfgd.org/), the advanced parameters are the significance level of 0.01 and the minimum gene number for each analyzed term of 3.

## Supplementary information

**Additional file 1: Figure S1.** QTL-allele matrices of the significant SNPLDBs associated with BN (A), BW(B) and LP(C), respectively.

**Additional file 2: Table S1.** Phenotypic distribution range of three yield-related traits among 315 upland cotton accessions.

**Additional file 3: Table S2.** The correlation analysis among three yield-related traits.

**Additional file 4: Table S3.** Analysis of variance of three yield-related traits in four planting environments.

**Additional file 5: Table S4.** The significant SNPLDB loci associated with BN, BW and LP, respectively.

**Additional file 6: Table S5.** A total of 115 genes annotated in the six adjacent genome fragments of the stable SNPLDB loci.

**Additional file 7: Table S6.** Gene names expressed mainly in fibers or ovules of upland cotton by two RNA-seq data.

**Additional file 8: Table S7.** The GO results of 16 potential candidate genes controlling target traits.

**Additional file 9: Table S8.** The KEGG results of 16 potential candidate genes controlling target traits.

**Additional file 10: Table S9.** The SNP loci associated with yield-related traits have been detected via GWAS method in upland cotton.

**Additional file 11: Table S10.** Information of the 315 upland cotton accessions.

**Additional file 12: Table S11.** The elastic GSC matrix and GSC eigenvectors.

## Data Availability

The sequence read data from SLAF-seq analysis for the sequenced upland cotton lines are available in the Sequence Read Archive (http://www.ncbi.nlm.nih.gov/bioproject/PRJNA314284/).

## Abbreviations

ANOVA: Analysis of variance
BN: Boll number
BSH: Broad-sense heritability
BW: Boll weight
CDS: Coding sequence
DPA: Days post anthesis
FPKM: Fragments per kilobase of transcript per million fragments mapped reads
FW: Fiber weight
GLM: Generalized Linear Model
GO: Gene ontology
GSC: Genetic similarity coefficient
GWAS: Genome-wide association study
KEGG: Kyoto encyclopedia of genes and genomes
LD: Linkage disequilibrium
LP: Lint percentage
LW: Lint weight
MAF: Minor allele frequency
ML-GWAS: Multi-locus GWAS
MLM: Mixed linear model
SA: Association analysis
QTL: Quantitative trait loci
SLAF-seq: Specific-locus amplified fragment sequencing
SNP: Single nucleotide polymorphism
SNPLDBs: SNP linkage disequilibrium blocks
SL-GWAS: Single-locus GWAS
PCA: Principal components analysis
PCs: Principal components
RTM-GWAS: Restricted two-stage multi-locus multi-allele GWAS
