# Supplementary figures and images for: An RTM-GWAS procedure reveals the QTL alleles and candidate genes for three yield-related traits in upland cotton
Source: BMC Plant Biol. 2020 Sep 7;20:416. doi: 10.1186/s12870-020-02613-y (PMC7487830; doi:10.1186/s12870-020-02613-y)

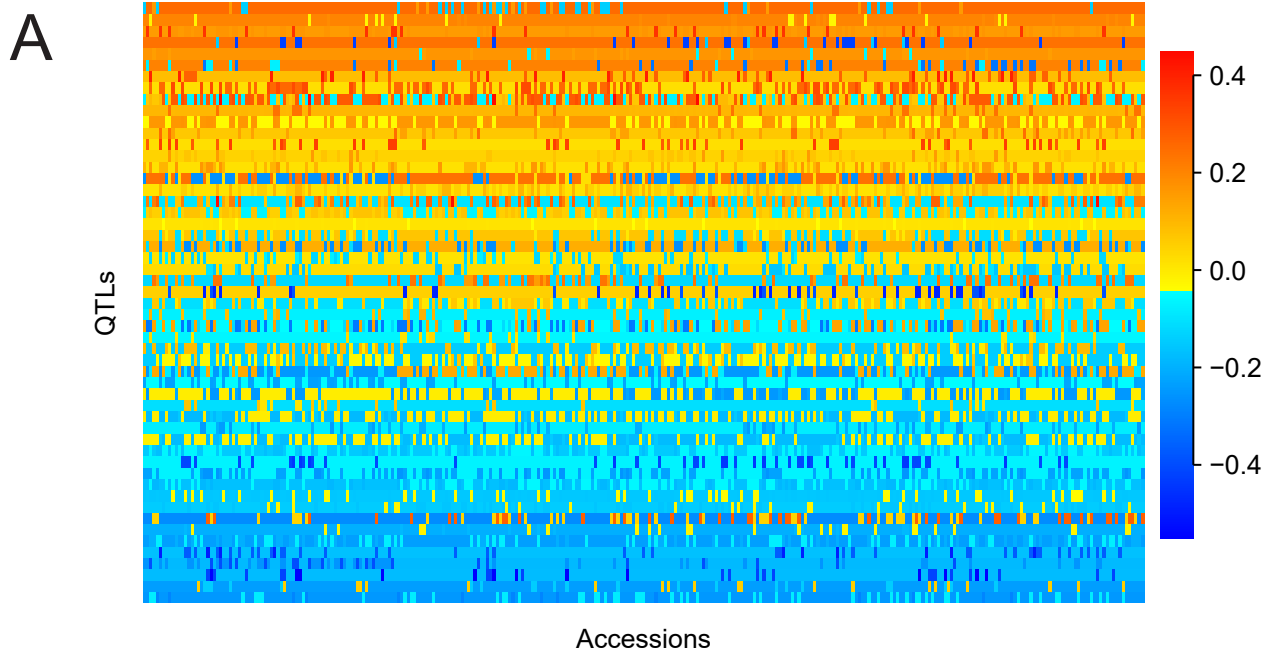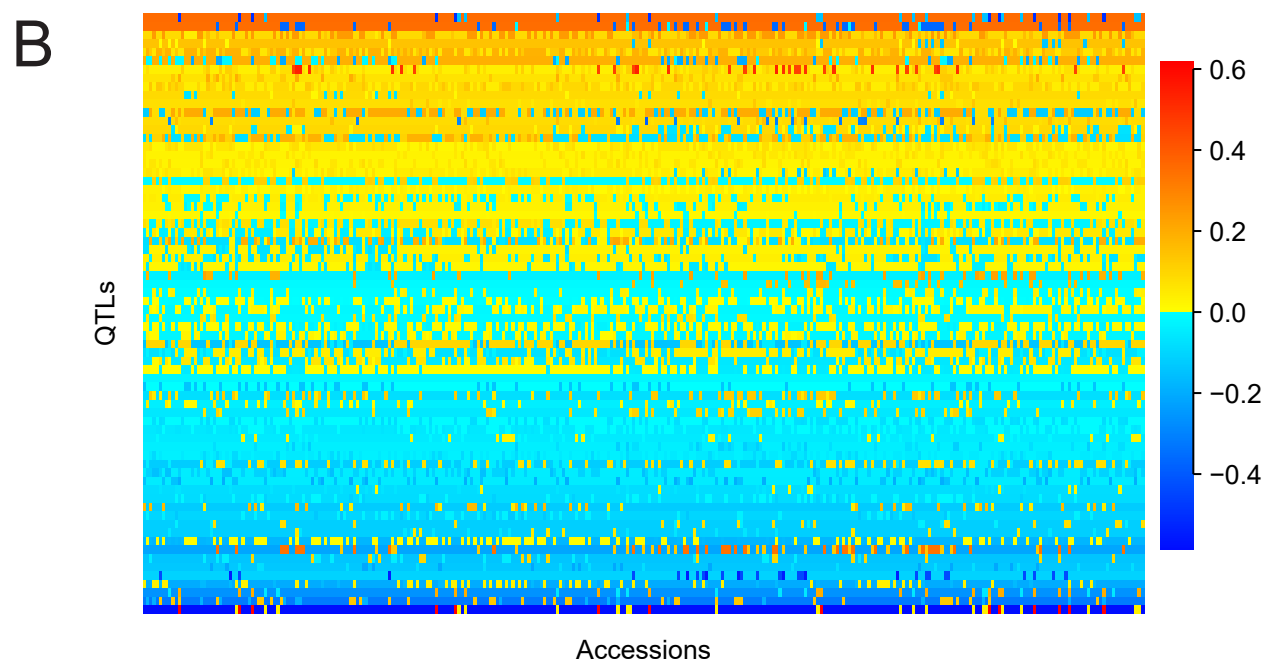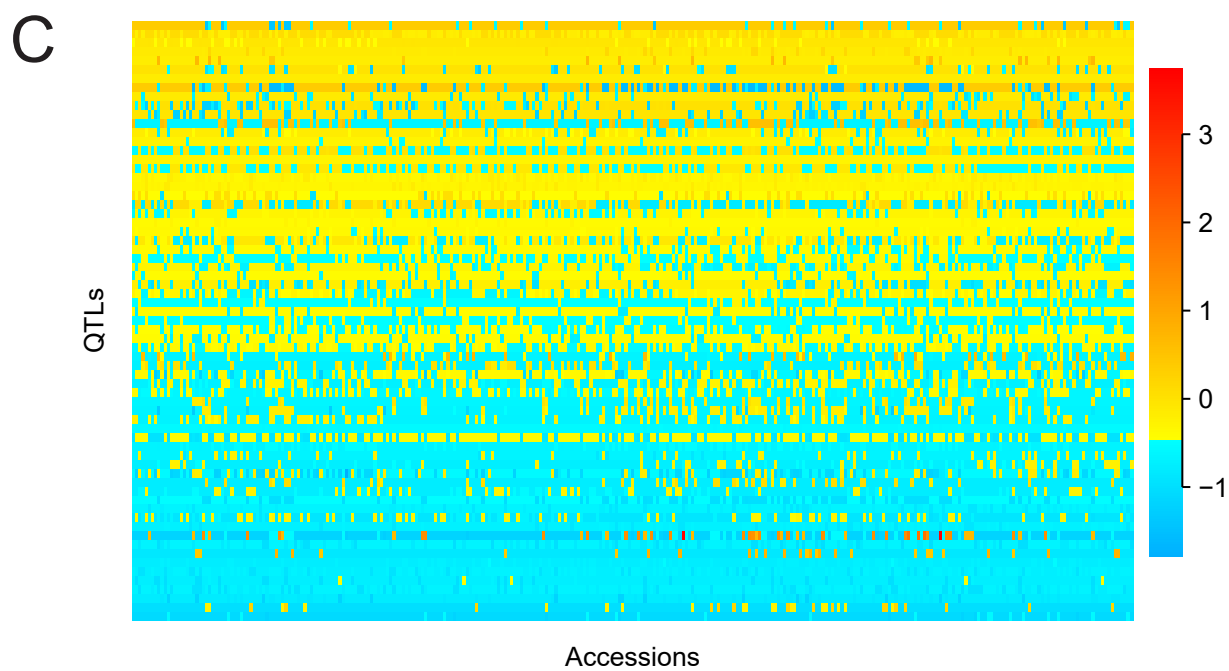

Supplement: Supplementary file 1 — Additional file 1: Figure S1. QTL-allele matrices of the significant SNPLDBs associated with BN (A), BW(B) and LP(C), respectively. [file 12870_2020_2613_MOESM1_ESM.pdf]
